# Molecular characterization of oriental eyeworm (*Thelazia callipaeda*) detected from raccoon (*Procyon lotor*) and Japanese raccoon dog (*Nyctereutes viverrinus*) in Kanto region, Japan

**DOI:** 10.1186/s13071-023-05736-x

**Published:** 2023-03-30

**Authors:** Kandai Doi, Toshihiro Tokiwa, Miyu Imoto, Shyun Chou, Fumiaki Yamasaki, Takuya Kato, Shin-ichi Hayama

**Affiliations:** 1 Laboratory of Wildlife Medicine, Nippon Veterinary and Life Science University, Kyonancho, Musashino, Tokyo, Japan; 2 Forestry and Forest Products Research Institute, Matsunosato, Tsukuba, Ibaraki Japan; 3 Laboratory of Veterinary Parasitology, Nippon Veterinary and Life Science University, Kyonancho, Musashino, Tokyo, Japan

**Keywords:** Oriential eyeworm, *Thelazia callipaeda*, Invasive species, Japan, Raccoon, Raccoon dog, Zoonosis

## Abstract

**Background:**

The oriental eyeworm *Thelazia callipaeda* (Spirurida: Thelaziidae) is an emerging parasitic ocular nematode of carnivores and humans. In domestic animals and humans, the infection causes varying degrees of inflammation and lacrimation, and wild carnivores represent an important reservoir. In this study we examined the infection status and molecular characterization of *T. callipaeda* in two urban carnivores, raccoons *Procyon lotor* and wild Japanese raccoon dogs *Nyctereutes viverrinus*, in the Kanto region of Japan.

**Methods:**

From January 2020 to December 2021, 193 carcasses including 178 raccoons and 15 raccoon dogs were examined for the presence of worms in the eye. The worms from infected animals (one worm per host) were morphologically identified as *T. callipaeda.* Worms (1–5 worms per host) were subjected to genetic analysis using mitochondrial cytochrome *c* oxidase subunit I gene sequences.

**Results:**

The prevalence of *T. callipaeda* in raccoons and Japanese raccoon dogs was 20.2% (36/178) and 13.3% (2/15), respectively. The *cox1* sequences from 56 worms from 38 animals revealed three haplotypes (h9, h10, and h12). Analysis of multiple worms for five raccoons showed co-infection of two different haplotypes (h9 and h10) in a single host. Comparing our data with published sequences, three sequences obtained from raccoons and raccoon dogs shared the same haplotypes as those reported in humans, dogs, and cats in Japan.

**Conclusions:**

Our findings show a high prevalence of *T. callipaeda* in raccoons, suggesting that this invasive carnivore species serves as an important natural reservoir of *T. callipaeda* in the Kanto region of Japan, an area with the highest human population of the country.

**Graphical Abstract:**

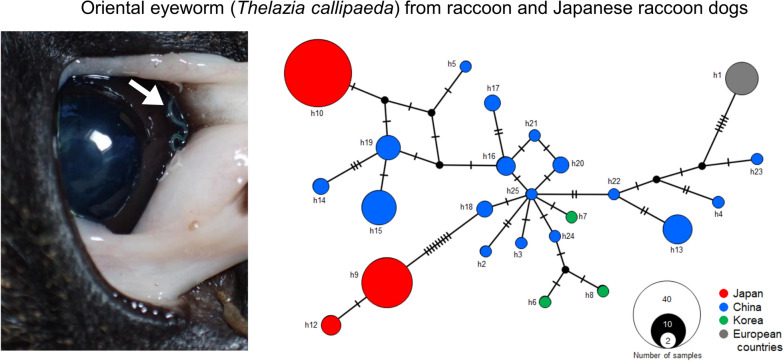

**Supplementary Information:**

The online version contains supplementary material available at 10.1186/s13071-023-05736-x.

## Background

The Oriential eyeworm, *Thelazia callipaeda* Ralliet and Henry, 1910, is a parasitic nematode that infects the eyes of wildlife, domestic animals, and humans [1, 2]. Adult *T. callipaeda* live in the conjunctival sac, and ovoviviparous *T. callipaeda* females release L1 in lacrimal secretions [3–6]. These L1 are ingested by fruit flies of the genera *Phortica* and *Amiota* (Diptera: Drosophilidae) when they feed on tears and conjunctival fluids. Ingested L1 grow into L3 in a fly and are transmitted to the eyes of susceptible vertebrate hosts [4, 6].

Human and canine thelaziosis caused by *T. callipaeda* has been reported in many countries in recent years. Thelaziosis appears to be particularly prevalent in European countries and the Russian Far East, where the parasite has been detected in wild animals including gray wolves *Canis lupus*, European rabbits *Oryctolagus cuniculus,* golden jackals *Canis aureus*, common raccoon dogs *Nyctereutes procyonoides*, brown bears *Ursus arctos*, Asian black bears *Ursus thibetanus*, lynx *Lynx lynx*, sable *Martes zibellina*, beech marten *Martes foina*, and European wildcat *Felis silvestris*, suggesting a rapid expansion of the parasite [7–15]. In Japan, the first report of *T. callipaeda* was on Kyusyu, located in the southwest portion of the Japanese island chain, and the worms have been sporadically reported since the 1950s in humans and domestic dogs and cats [16–18]. It is generally believed that *T. callipaeda* spread to the eastern part of Japan, including the Kanto region, after the 1970s, where it has been detected in humans, dogs, cats, and Japanese raccoon dogs [19, 20].

The raccoon *Procyon lotor* is a medium-sized carnivore originally native to North and Central America. Raccoons were first introduced in Japan in the late 1960s [21], and they are now one of the most problematic non-native species in Japan. The Japanese government investigated feral raccoon distribution in 2006, confirming their dispersion across most of the prefectures of Japan. Feral raccoons are now considered an invasive species and prey on endangered amphibians and crustaceans, damage crops and houses, and compete for resources with sympatric mammals [21]. Meanwhile, the Japanese raccoon dog *Nyctereutes viverrinus* is an endemic medium-sized carnivore found throughout Japan [22]. This species lives in forested areas, but is also frequently found in agricultural land and urban areas [23]. In urban and suburban environments, the distributions of raccoons and raccoon dogs overlap, and both species may harbor pathogens, including parasites [7, 24, 25].

In Japan, although infection with *T. callipaeda* in domestic animals and humans has been reported sporadically in many areas [17–20, 26], the prevalence of *T. callipaeda* in wildlife has not been thoroughly investigated. Here, we examined the infection status of *T. callipaeda* in feral raccoons and wild raccoon dogs in the Kanto region of Japan. We also observed the genetic differences between *T. callipaeda* strains in Japan and its neighboring countries.

## Methods

### Study area

**Fig. 1:**
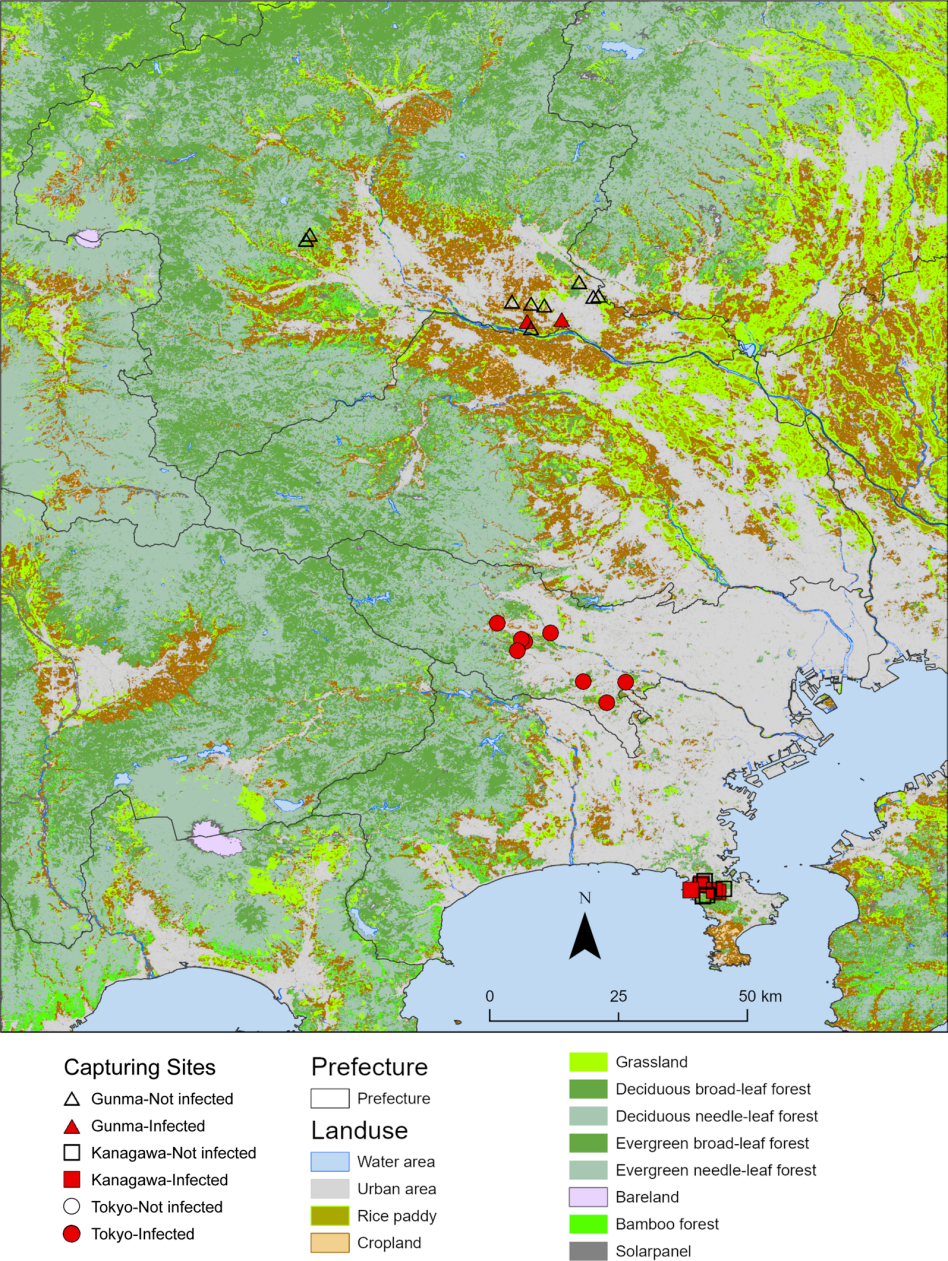
Geographical distribution of *Thelazia callipaeda*-infected animals (red marker) and non-infected animals

This survey was conducted in the Kanto region, which is located in the southeastern part of the main island of Japan and is dominated by the Kanto Plain, the largest plain in Japan (Fig. 1). The Kanto region has a humid subtropical climate, and the four seasons are sharply delineated. This region is the most populous and urbanized in Japan. The native carnivorous species, Japanese weasel *Mustela itatsi*, Japanese marten *Martes melampus*, Japanese badger *Meles anakuma*, Japanese red fox *Vulpes vulpes japonica*, and Japanese raccoon dogs have been observed only in the suburbs of the Kanto region, where woodlands and grasslands remain. Invasive carnivore species, such as raccoons and masked palm civets *Paguma larvata*, have been observed in residential areas [27].

### Animal samples

From January 2020 to December 2021, 193 carcasses, including of 178 raccoons and 15 Japanese raccoon dogs, were examined for the presence of worms in the eye. All animals were caught by licensed hunters using box traps (Havahart, Model 1089; Woodstream, PA) and killed using a combination of xylocaine and pentobarbital sodium or carbon dioxide gas. These carcasses were bagged in two or three layers of plastic bags immediately after death, sent to Nippon Veterinary and Life Science University under freezing or frozen conditions, and subsequently necropsied. The age of all the animals was estimated according to the extent of tooth eruption. We classified the raccoons into two groups: pre-weaned juvenile raccoons (< 5 months old) and weaned subadult and adult raccoons (≥ 5 months old), hereafter referred to as “adult raccoons” [28]. Japanese raccoon dogs were divided into two groups: juvenile raccoons (< 6 months old) and subadult and adult raccoons (≥ 6 months old), hereafter referred to as “adult raccoon dogs” [29, 30]. Detailed information regarding the place of capture, as well as the weight, estimated age by stage, and sex of the animals is included in Table 1.

**Table 1:** Infected animals and *Thelazia callipaeda* haplotypes

Host ID	Host	Localities	Date (day-month-yrea)	Sex	Body weight (g)	Age class	*T. callipaeda*	
							Worm burden	*cox1* haplotypes (number of worms analyzed)
MIO1	*Pl*	Hayama, Kanagawa	30-Jan-20	M	5430	Adult	5	h10 (1)
MIO2	*Nv*	Ohta, Gunma	18-Mar-20	M	3910	Adult	7	h9 (1)
MIO3	*Pl*	Fussa, Tokyo	07-May-20	F	4380	Adult	3	h10 (1)
MIO4	*Pl*	Hayama, Kanagawa	26-Sep-20	M	4375	Juvenile	1	h10 (1)
MIO5	*Pl*	Hayama, Kanagawa	24-Nov-20	M	3695	Adult	3	h10 (1)
MIO6	*Pl*	Fussa, Tokyo	15-Apr-21	M	5260	Adult	6	h10 (1)
MIO7	*Pl*	Hayama, Kanagawa	23-Apr-21	F	4375	Adult	1	h10 (1)
MIO8	*Pl*	Machida, Tokyo	09-Jun-21	F	5380	Adult	2	h9 (1)
MIO9	*Pl*	Tama, Tokyo	10-Jun-21	M	7210	Adult	1	h9 (1)
MI10	*Pl*	Tama, Tokyo	10-Jun-21	M	5515	Adult	1	h9 (1)
MI11	*Pl*	Machida, Tokyo	08-Jun-21	F	5880	Adult	10	h9 (1), h10 (4)
MI12	*Pl*	Tama, Tokyo	08-Jun-21	M	5925	Adult	2	h12 (1)
MI13	*Pl*	Machida, Tokyo	30-Jul-21	F	5895	Adult	1	h10 (1)
MI14	*Pl*	Tama, Tokyo	29-Jul-21	M	5915	Adult	7	h10 (1)
MI15	*Nv*	Ohta, Gunma	05-Jan-21	M	3495	Adult	1	h10 (1)
MI16	*Pl*	Hayama, Kanagawa	22-Jun-21	F	5600	Adult	9	h9 (1)
MI17	*Pl*	Hachioji, Tokyo	03-Aug-21	M	2030	Juvenile	4	h10 (1)
MI18	*Pl*	Tama, Tokyo	03-Aug-21	F	4815	Adult	14	h9 (1), h10 (3)
MI19	*Pl*	Machida, Tokyo	28-Jul-21	M	2740	Juvenile	23	h9 (1), h10 (3)
MI20	*Pl*	Machida, Tokyo	28-Jul-21	M	2845	Juvenile	44	h9 (1), h10 (3)
MI21	*Pl*	Hachioji, Tokyo	30-Jul-21	M	2920	Juvenile	9	h10 (1)
MI22	*Pl*	Fussa, Tokyo	06-Jul-21	M	6410	Adult	1	h10 (1)
MI23	*Pl*	Machida, Tokyo	31-Aug-21	M	3170	Juvenile	8	h10 (1)
MI24	*Pl*	Machida, Tokyo	31-Aug-21	M	3455	Juvenile	8	h9 (1)
MI25	*Pl*	Machida, Tokyo	31-Aug-21	F	3545	Juvenile	2	h9 (1)
MI26	*Pl*	Machida, Tokyo	31-Aug-21	F	3245	Juvenile	5	h9 (1)
MI27	*Pl*	Machida, Tokyo	01-Sep-21	F	5125	Adult	2	h9 (1)
MI28	*Pl*	Machida, Tokyo	08-Sep-21	F	6260	Adult	1	h12 (1)
MI29	*Pl*	Machida, Tokyo	08-Sep-21	M	4450	Juvenile	24	h9 (4), h10 (1)
MI30	*Pl*	Hachioji, Tokyo	15-Sep-21	F	5115	Adult	7	h10 (1)
MI31	*Pl*	Hayama, Kanagawa	27-Aug-21	M	5135	Adult	1	h10 (1)
MI32	*Pl*	Hachioji, Tokyo	05-Oct-21	F	5455	Adult	9	h10 (1)
MI33	*Pl*	Machida, Tokyo	06-Oct-21	F	6160	Adult	2	h10 (1)
MI34	*Pl*	Hachioji, Tokyo	20-Oct-21	M	6330	Adult	5	h10 (1)
MI35	*Pl*	Hachioji, Tokyo	21-Oct-21	F	4810	Juvenile	4	h9 (1)
MI36	*Pl*	Hachioji, Tokyo	21-Oct-21	F	5920	Adult	6	h9 (1)
MI37	*Pl*	Fussa, Tokyo	20-Oct-21	M	7155	Adult	1	h10 (1)
MI39	*Pl*	Fussa, Tokyo	11-Oct-21	M	5735	Adult	1	h9 (1)

*Pl*: *Procyon lotor*; *Nv*: *Nyctereutes viverrinus*; M: male; F: female

### GIS mapping

The geographical distribution of *T. callipaeda*-infected and non-infected animals was displayed on the maps. ArcGIS Pro software (ESRI, Inc., West Redland, CA, USA) was used to project the capture locations of the examined animals.

### Nematode collection

The third eyelid and conjunctival sacs of the animals were carefully examined for the presence of adult eyeworms. The worms were collected mechanically using sterile swabs and stored in 70% ethanol. The worms were mounted on slides in 50% glycerol and 40% ethanol solutions, observed using a BX53 microscope (Olympus, Tokyo), and identified as *T. callipaeda* according to morphological keys [31].

### Molecular analysis

A total of 55 adults of *T. callipaeda* collected from the infected animals (1–5 worms per host) were used for molecular analysis. Genomic DNA was extracted from the middle part of each worm using a QIAGEN DNA mini kit (Qiagen, Germany), according to the manufacturer’s instructions. Double-distilled water was used as the negative control.

Partial fragments of mitochondrial *cox1* were amplified using the specific primers NTF (5′-TGATTGGTGGTTTTGGTAA-3′) and NTR (5′-ATAAGTACGAGTATCAATATC-3′) [32]. PCR was performed using 20 μl reaction volumes, each containing 0.2 μl TaKaRa ExTaq polymerase (TaKaRa, Japan), 2 μl 10 × buffer, 1.6 μl dNTPs (2.5 mM of each), 0.2 μl of each primer (50 μM), 1.0 μl template, and 14.8 μl double-distilled water. The thermocycling conditions were as follows: initial denaturation at 95 °C for 5 min followed by 35 cycles of denaturation at 94 °C for 30 s, annealing at 40 °C for 30 s, and extension at 72 °C for 1 min. The final extension was performed at 72 °C for 10 min, followed by a holding step at 4 °C. PCR products were separated by electrophoresis on 1.5% agarose gels and visualized under an LED transilluminator after staining with GRGreen (BioCraft, Japan). The sizes of the PCR products were estimated by comparison with a 100-bp DNA ladder (Maestrogen, Taiwan). The PCR products were purified using ExoSAP-IT (Macrogen Corp., Japan) and analyzed using an ABI 3730xl DNA analyzer (Thermo Fisher Scientific, USA) with the same PCR primers.

Sequence similarity was studied using the BLASTN program available on the National Center for Biotechnology Information database (http://www.ncbi.nlm.nih.gov/Blast.cg). Multiple sequence alignment was performed using MAFFT ver. 7.505 with the Q-INS-I option [33]. For phylogenetic analysis, a 642-bp dataset generated from multiple alignments of 117 *cox1* sequences (62 from the public database and 55 determined in this study) was used for haplotype and network analyses (Additional files 1 and 2). Definition and numbering of haplotypes were based on previous studies [31, 34]. Haplotype networks were generated using the medium-joining network algorithm in PopART ver. 1.7 [35, 36]. The haplotype diversity and nucleotide diversity at each site were calculated using DnaSP ver. 6.12.03 [37].

## Results

### Prevalence and geographical distribution of *Thelazia callipaeda*

A total of 233 *T. callipaeda* individuals were collected from 36 raccoons (Table 1). The mean worm burden, SD and range of parasite number (in parentheses) were 6.5 ± 8.4 (1–44). Thirty infected individuals (16 males and 14 females) were captured in Tokyo, and six individuals (4 males and 2 females) were captured in Kanagawa. Eight worms were collected from two male Japanese raccoon dogs (Table 1).

In raccoons, the overall infection rate was 20.2% (36/178; 95% CI 15.0–26.8) (Table 2). Juvenile male raccoons showed the highest infection rate of 36.4% (8/22; 95% CI 19.6–57.1), followed by adult female raccoons at 23.5% (12/51; 95% CI 13.9–36.9), adult male raccoons at 14.9% (13/87; 95% CI 8.8–24.0), and juvenile female raccoons at 11.1% (3/18; 95% CI 5.2–40.2). The overall infection rate in Japanese raccoon dogs was 13.3% (2/15; 95% CI 2.5–39.1) (Table 3). In Japanese raccoon dogs, *T. callipaeda* was detected only in adult male individuals (25.0%, 2/8; 95% CI 6.3–59.9).

**Table 2:** Prevalence of *Thelazia callipaeda* in wild raccoon in Kanto region, Japan

Variable	No. examined	No. positive	Prevalence	
	*n*	*n*	%	95% CI
Prefecture				
Tokyo	124	30	24.2	17.5–32.5
Kanagawa	44	6	11.6	6.0–27.0
Gunma	10	0	0	0–32.1
Sex				
Male	109	20	18.3	12.2–26.8
Female	69	16	23.2	14.8–34.5
Age				
Adult	138	25	18.1	12.6–25.5
Juvenile	40	11	27.5	16.1–43.0
Total	178	36	20.2	15.0–26.8

**Table 3:** Prevalence of *Thelazia callipaeda* in wild Japanese raccoon dogs (*Nyctereutes viverrinus*) in Gunma Prefecture, Japan

Variable	No. examined	No. positive	Prevalence	
	*n*	*n*	%	95% CI
Sex				
Male	8	2	25.0	6.3–59.9
Female	7	0	0	0–40.4
Age				
Adult	14	2	14.3	2.8–41.2
Juvenile	1	0	0	0–83.2
Total	15	2	13.3	2.4–39.1

### Molecular identification

Partial *cox1* sequences (667 bp) were also determined separately for each individual worm, and sequence alignments differentiated the three haplotypes. Nineteen worms from 16 raccoons and one worm from a Japanese raccoon dog shared one sequence type, which was 100% identical to *T. callipaeda* sequences reported from humans in the Okayama Prefecture, Japan (640/640 bp; accession no. AB538283) and a dog in Tokyo, Japan (660/660 bp; accession no. AB852544). Zhang et al. [34] referred to the former sequence reported from Okayama as haplotype h9 and the latter reported from Tokyo as h11. However, since these sequences are indistinguishable from each other (635/635 bp), we have unified them and identified as h9. The second sequence, obtained from 32 worms from 22 individual raccoons and one worm from a Japanese raccoon dog, was identical to the sequence reported in humans in Kumamoto (596/569 bp; accession no. LC565618) and Oita Prefectures (585/585 bp; accession no. LC565619) and dogs (660/660 bp; accession nos. AB852543, AB852546–AB852548, AB852550) and a cat (660/660 bp, accession no. AB852549) in Tokyo, Japan. The third sequence type, obtained from two worms and two raccoons, was identical to a sequence recorded from a dog in Tokyo (660/660 bp; accession no. AB852545). The second and third haplotypes were formerly known as h10 and h12, respectively [34]. In the five raccoons analyzed, with 4–5 worms per individual, all had mixed infection with h9 and h10 haplotypes (Table 1). Representative sequences were deposited in the DNA Data Bank of Japan under accession numbers LC46896–LC46898.

Multiple alignment of the available sequences and sequences obtained in this study revealed 24 haplotypes (Table 4); 3 from Japan (h9, h10, and h12), 17 from China, 3 from Korea, and 1 from European countries (Germany, Greece, The Netherlands, Italy, Romania, Slovakia, and Serbia). Upon comparison of haplotypes recorded from Japan and China, which had larger sample sizes, both haplotype and nucleotide diversity were higher in China.

**Table 4:** Genetic diversity of *cox1* sequence from *Thelazia callipaeda*

Population	Sample size	No. of haplotypes	No. of substitution sites	Haplotype diversity (SD)	Nucleotide diversity	Fu’s *Fs* statistic	Tajima’s *D* (*P* < 0.001)
Japan	64	3	16	0.507 (0.042)	0.011	17.864	3.389
China	42	17	21	0.904 (0.027)	0.067	− 4.365	− 0.405
Korea	3	3	5	1.000 (0.272)	0.005	–	–
European countries	9	1	0	0	0	–	–

**Fig. 2:**
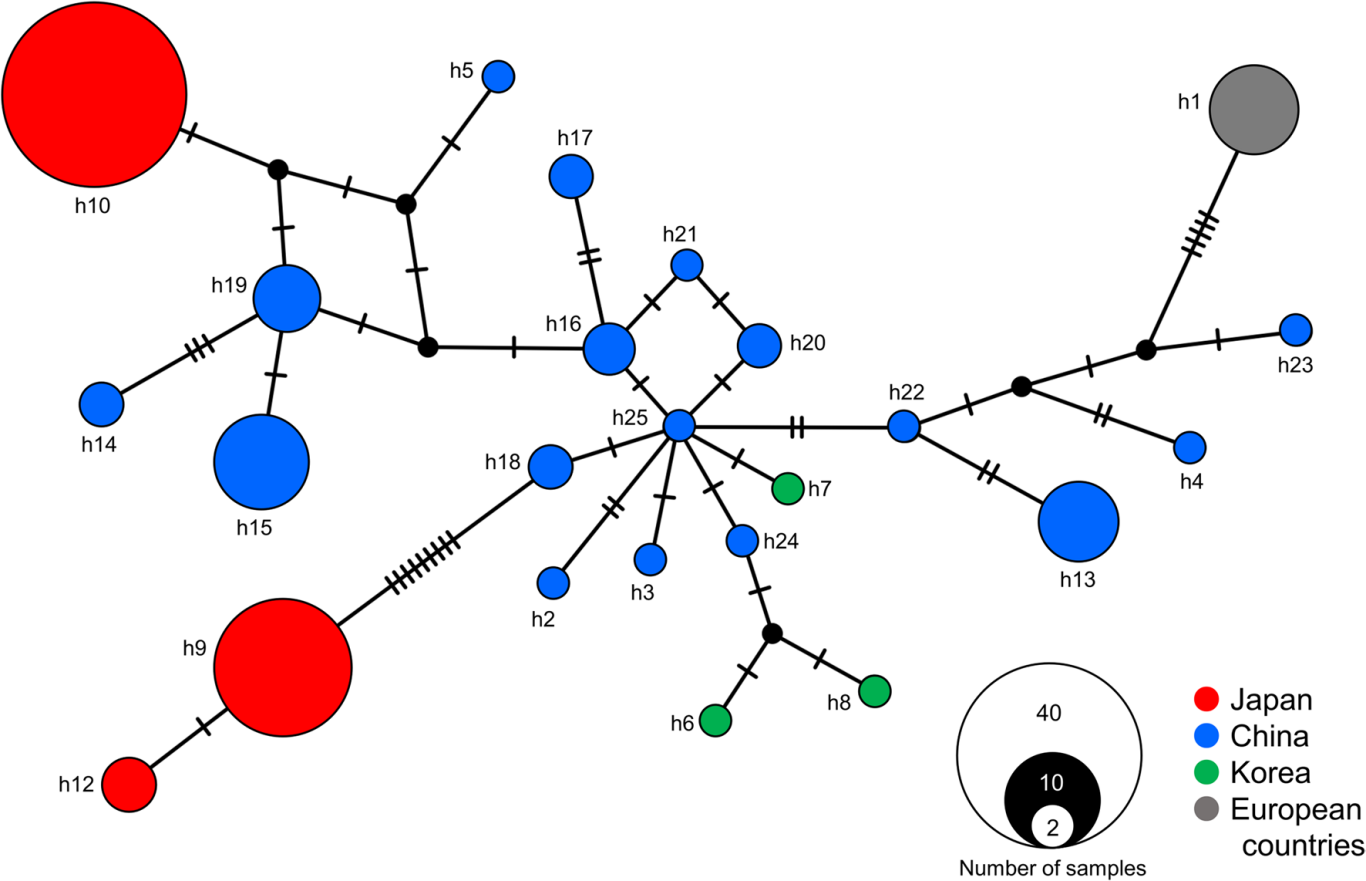
Haplotype network of *cox1* haplotypes found in *Thelazia callipaeda* from Asian and European countries. Each cycle represents a haplotype with the size proportional to its sampling frequency. Colors represent the locality of the host reported. Black dots and bars on lines represent hypothetical haplotypes and the number of additional mutation steps between two haplotypes

A median-joining network of *T. callipaeda* haplotypes revealed that the Chinese haplotypes were centrally located in the network and were accompanied by some haplotypes reported from China and Korea (Fig. 2). The three Japanese haplotypes and the European haplotype (h1) were located outside this group and were not connected directly to each other, except for h9 and h12.

## Discussion

To the best of our knowledge, *T. callipaeda* has not been detected in raccoons in North America or any countries where raccoons have been introduced [7]. Meanwhile, *T. callipaeda* has reportedly been detected in multiple individuals of common raccoon dogs in Russia [9, 10], but only one case in Japanese raccoon dogs has been reported in Japan [38]. In this study, 20.2% of raccoons and 13.3% of Japanese raccoon dogs in the Kanto region of Japan were infected with *T. callipaeda*. Few studies have examined the infection rate of *T. callipaeda* in animals in Japan: a survey in 1981 in the Hyogo Prefecture found a prevalence of 0.2% (1/521) in outdoor dogs [26]; a survey in 1992 of outdoor dogs and cats in the Hiroshima Prefecture found a prevalence of 1.0% (5/500) and 0.4% (2/250), respectively [39]; a survey in 2009 of outdoor dogs and cats in Tokyo found 4.0% (4/100) and 2.0% (2/100) prevalence, respectively [20]. Compared to these facts, this study reports the first investigation of thelaziosis in raccoons, and the infection rates in raccoons and sympatric Japanese raccoon dogs are higher than other regions of Japan, suggesting that these animals are more involved in the transmission of *T. callipaeda* in the Kanto region of Japan.

Genetic analysis showed that haplotype diversity in *T. callipaeda* collected from Japan was lower than among that from the continental origin, as Japan has only three haplotypes of *T. callipaeda* (h9, h10, and h12). These three Japanese haplotypes appear to have low host specificity, as h9 has been reported in humans, domestic dogs, raccoons, and Japanese raccoon dogs; h10 has been reported in humans, dogs, cats, raccoons, and Japanese raccoon dogs; and h12 has been reported in dogs and raccoons. Of these haplotypes, h9 and h12 are genetically related, whereas h10 has a separate lineage. The low number of haplotypes and the genetic diversity of *T. callipaeda* in Japan may be a result of its relatively recent introduction. A similar situation was observed during its initial introduction in Italy [40], followed by France [41] and Spain [42], where the same haplotype (h1) was detected. Future studies on the infection status of *T. callipaeda* and its haplotypes from other native Japanese carnivores, such as Japanese weasels, martens, badgers, and red foxes, are required.

Feral raccoons in Japan emerged after individuals escaped from zoos in the 1960s or were kept as exotic pets in the 1970s, and their distribution has spread from urban areas to suburban areas [21]. Thus, raccoons are now part of the urban wildlife of Japan and are found in residential areas, green spaces, and rivers in suburban areas. Raccoon dogs frequently visit residential areas but use woodlands and grasslands more frequently than raccoons [27]. The detection of *T. callipaeda* in raccoons and raccoon dogs and the identical molecular characteristics between our samples and the samples detected in humans, dogs, and cats indicate that this zoonotic nematodes is able to infect and spread between many species frequently found in urban areas in Japan.

## Conclusion

Feral raccoons and wild Japanese raccoon dogs in the Kanto region of Japan showed high *T. callipaeda* infection rates and the same *T. callipaeda* with haplotypes identical to those identified for the parasites found in humans, dogs, and cats. The results of this study indicate that invasive raccoons are additional definitive hosts for *T. callipaeda* in Japan. The feral raccoon is a successful urban wildlife species in multiple countries and thus may play a role in transmitting the oriental worm to domestic dogs, cats, and possibly to humans.

## Supplementary Information

**Additional file 1: Dataset S1.** Dataset of *cox1* sequences of *Thelazia callipaeda* in text and fasta format*.*

**Additional file 2: Table S2.** Geographical origin, host, GenBank/DDBJ/EMBL accession nos., and haplotype name of the *Thelazia cox1* sequences.

## Data Availability

The manuscript and its supporting information files contain all necessary information.

## Abbreviations

CI: Confidence interval
*cox1*: Cytochrome *c* oxidase subunit I gene
L1: First stage larvae
L3: Third stage larvae
PCR: Polymerase chain reaction
SD: Standard deviation
